# Recent Progress in Hybrid Biocomposites: Mechanical Properties, Water Absorption, and Flame Retardancy

**DOI:** 10.3390/ma13225145

**Published:** 2020-11-15

**Authors:** Mohsen Bahrami, Juana Abenojar, Miguel Ángel Martínez

**Affiliations:** Materials Science and Engineering and Chemical Engineering Department, University Carlos III de Madrid, 28911 Leganes, Spain; abenojar@ing.uc3m.es (J.A.); mamc@ing.uc3m.es (M.Á.M.)

**Keywords:** biocomposites, green polymers, hybrid composites, natural fibers, mechanical properties

## Abstract

Bio-based composites are reinforced polymeric materials in which one of the matrix and reinforcement components or both are from bio-based origins. The biocomposite industry has recently drawn great attention for diverse applications, from household articles to automobiles. This is owing to their low cost, biodegradability, being lightweight, availability, and environmental concerns over synthetic and nonrenewable materials derived from limited resources like fossil fuel. The focus has slowly shifted from traditional biocomposite systems, including thermoplastic polymers reinforced with natural fibers, to more advanced systems called hybrid biocomposites. Hybridization of bio-based fibers/matrices and synthetic ones offers a new strategy to overcome the shortcomings of purely natural fibers or matrices. By incorporating two or more reinforcement types into a single composite, it is possible to not only maintain the advantages of both types but also alleviate some disadvantages of one type of reinforcement by another one. This approach leads to improvement of the mechanical and physical properties of biocomposites for extensive applications. The present review article intends to provide a general overview of selecting the materials to manufacture hybrid biocomposite systems with improved strength properties, water, and burning resistance in recent years.

## 1. Introduction

**Figure 1:**
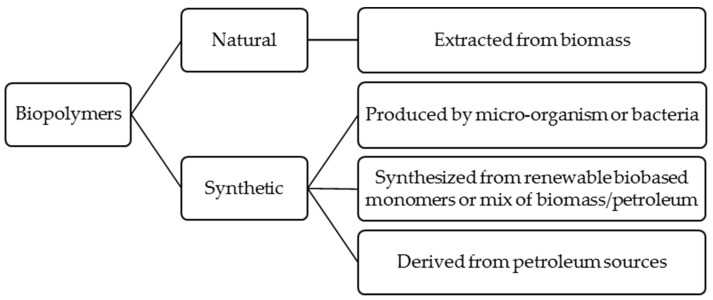
Origins of biopolymer materials [©MB, JA, MAM_UC3M2020].

Nowadays, global environmental safety, ecological concerns, recyclability, eco-efficiency, and economic factors are fundamental driving forces to increase the employment of bio-based materials [1,2]. The EU Bioeconomy Strategy [3] is one of the developed plans or policies to accelerate the UN Sustainable Development Goals (SDGs). This strategy focuses not only on shifting from fossil resources to renewable raw materials but also on recycling renewable bio-based raw materials and innovating in the production and consumption of materials [3]. In this regard, biocomposites are among the potential sectors for the bioeconomy. Renewable and sustainable biomaterials as an alternative for petroleum-based materials prevent generating carbon dioxide causing global warming. They would be a solution to alleviating the petroleum supplies’ uncertainty, which are about to decline shortly [4,5]. Within recent years, biopolymers or green polymers have received tremendous interest among biomaterials. The term biopolymer is a polymer of monomeric molecules derived from natural resources, including biological systems or living organisms. Moreover, biopolymers can be found in nature and daily life, such as natural rubber, starch, cotton, leather, wool, etc. [6,7,8]. Biopolymers are renewable, environmentally friendly, or/and biodegradable materials, which can be divided into two broad groups of natural and synthetic (Figure 1) with the following origins [9,10,11,12,13]:

- Natural biopolymers extracted from biomass (e.g., polysaccharides, proteins)
- Synthetic biopolymers produced by a micro-organism or bacteria (e.g., bacterial cellulose, polyhydroxyvalerate, polyhydroxybutyrate)
- Synthetic biopolymers synthesized from renewable bio-based monomers or mixed sources of biomass and petroleum (e.g., polylactic acid, aliphatic polyester, aliphatic-aromatic copolyesters)
- Biodegradable polymers that are derived from nonrenewable resources (petroleum sources) (e.g., polycaprolactone)

Despite the advantages of biopolymer materials, they have some limitations compared to petroleum-based materials, such as relatively low stiffness, tensile strength, permeability, and thermal stability [8,9]. Therefore, to modify biopolymers’ properties and commercial importance, “biopolymer composites” are tremendously explored by adding reinforcement materials within the micro or nanoscale [14,15,16,17,18]. These composites have a large assortment of applications in construction, medicine, electronics, packaging, and automotive sectors [19,20,21,22,23,24].

**Figure 2:**
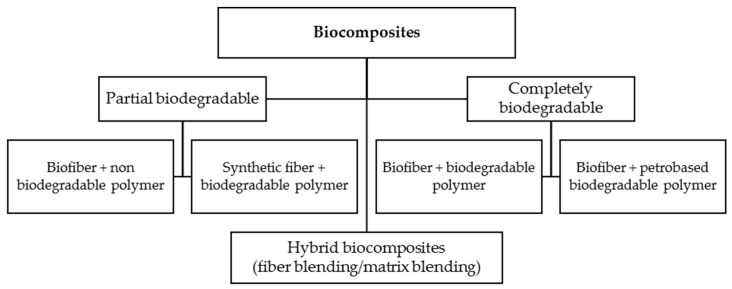
Classification of biocomposites. Adapted from [5].

Composite materials systems are made from one or more discontinuous reinforcing phases embedded in a continuous matrix phase. Biopolymer composites or biocomposites are referred to as composites in which at least one constituent is bio-based or biodegradable [25]. The focus of this review will be on the fibrous composites rather than laminate and particulate composites. Fibrous composites can be made of natural/biofiber and synthetic fiber. Biocomposite systems can be classified into partial biodegradable and complete biodegradable biocomposites on the basis of the matrix and fibers (Figure 2) [2,5,26]. In the complete biodegradable biocomposites, bio-fibers are employed with the matrices are made of biodegradable polymers such as renewable biopolymer matrices (e.g., cellulosic plastic, soy plastic, starch plastic) or petro-based biodegradable polymer matrices (e.g., aliphatic co-polyester, polyesteramides). However, in the partial biodegradable biocomposites, bio-based matrices are reinforced with synthetic fibers, or non-biodegradable polymers matrices such as traditional thermoplastic polymers (e.g., polypropylene, polyethylene) and thermoset polymers (e.g., epoxy, polyester) are reinforced with bio-fibers [5,26]. It is worth noting this classification of biocomposites does not estimate time or amount of degradation, which depends on many parameters, such as environment, temperature, and micro-organisms and can be defined by standard degradation tests. This classification emphasizes that if both fibers and matrix be 100% biodegradable materials, a composite will decompose at the end of its life and completely go back into the natural environment. Otherwise, if one of them be non-biodegradable, the composite doesn’t show full biodegradability properties. Recently, the blending of two or more polymers reinforced with one or more fibers that produce hybrid composites materials has become an effective way to manipulate biocomposites properties and maintain the balance among ecology-economy-technology. The present study reviews the recent progress of hybrid biocomposites in improving their mechanical properties, water, and burning resistance.

## 2. Hybridization

The concept of hybridization can be found in various fields, such as mechanics, polymer and chemistry, metallurgy, physics, science and technology, and energy fuels. Regardless of the research area, this process’s main objective is to combine three or more materials to achieve improved performance or properties compared to each substance for a proposed application [27]. In the field of composite materials, by incorporating two or more filler/fiber into a matrix material, the disadvantages of one type of reinforcement can be reduced by another one [28]. For example, by hybridization of natural fibers with any synthetic fibers, although the main benefits of natural fiber composites such as biodegradability, easy handling and processing, and natural resources are being reduced, hybridization can decrease the cost, weight, and environmental concerns produced by the synthetic fiber composites effectively. Several publications scientifically prove that hybridization techniques can enhance the composites’ mechanical, thermal, and dynamic characteristics, such as minimizing water absorption and increasing flame retardancy [29,30,31]. The main parameters, which significantly affect the properties of the manufactured hybrid composite products, are as follows [27]:

- Materials selection (matrix and fiber), which depend mostly on the proposed application;
- Preparation technique, which depends on the fiber, matrix, and working conditions (viz. outdoors or indoors);
- Interaction between the fibers and matrix, which can be controlled by fiber treatment or the use of coupling agents.

**Figure 3:**
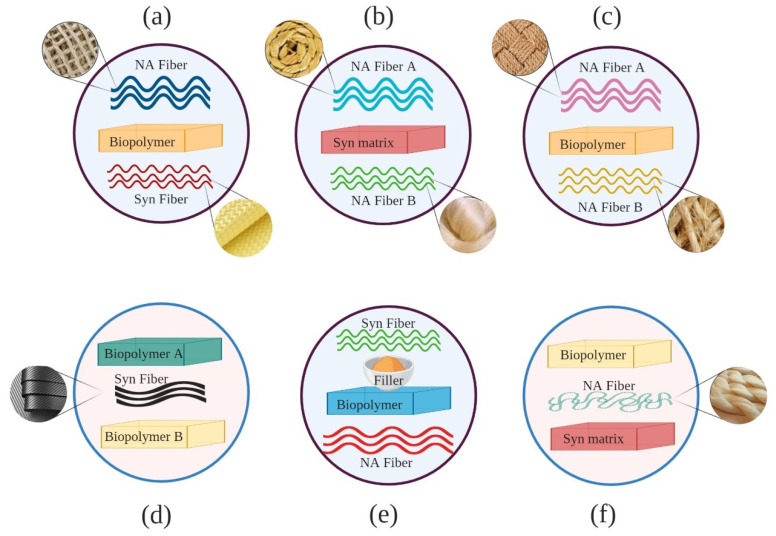
Some configurations of hybrid biocomposites (NA: natural, Syn: synthetic): (**a**) hybridization of natural and synthetic fibers with biopolymer matrix; (**b**) hybridization of two or more natural fibers with synthetic matrix; (**c**) hybridization of two or more natural fibers with biopolymer matrix; (**d**) hybridization of two or more biopolymer matrices with synthetic fiber; (**e**) hybridization of natural and synthetic fibers with fillers with biopolymer matrix (**f**) hybridization of biopolymer and synthetic matrices with natural fiber. [©MB, JA, MAM_UC3M2020].

Considering the mentioned parameters is significantly essential, since they could cause unexpected effects in the results. Many studies have reported adverse effects of hybridization due to the inappropriate selection of materials, processing techniques, a sequence of layers in a hybrid structure, and the loading arrangement. For instance, Sreekala et al. [32] reported a negative effect of oil palm empty fruit bunch (OPEFB) fiber hybridization on the tensile strength and tensile modulus at very low and high fiber volume fractions. Zweben [33] predicted that high elongation fibers might behave like crack arrestors on a micromechanical level when introduced in low elongation fiber composites. This behavior increases the strain level needed for fiber break propagation. Hariharan and Abdul Khalil [34], who hybridized oil palm fibers with glass fibers, also stated an undesired hybrid effect on the strength properties. Marom et al. [35] investigated positive or negative hybrid effects in hybrid composites as a positive or negative deviation of a specific mechanical property according to the rule-of-mixtures behavior. It is based on the weighted average of the characteristic properties of the distinct composites [36]. Based on biocomposites classification (Figure 2) and the concept of hybridization, different configurations of hybrid fiber-reinforced biocomposites could be manufactured as some of them are depicted in Figure 3.

## 3. Materials Selection

### 3.1. Fiber Selection

**Figure 4:**
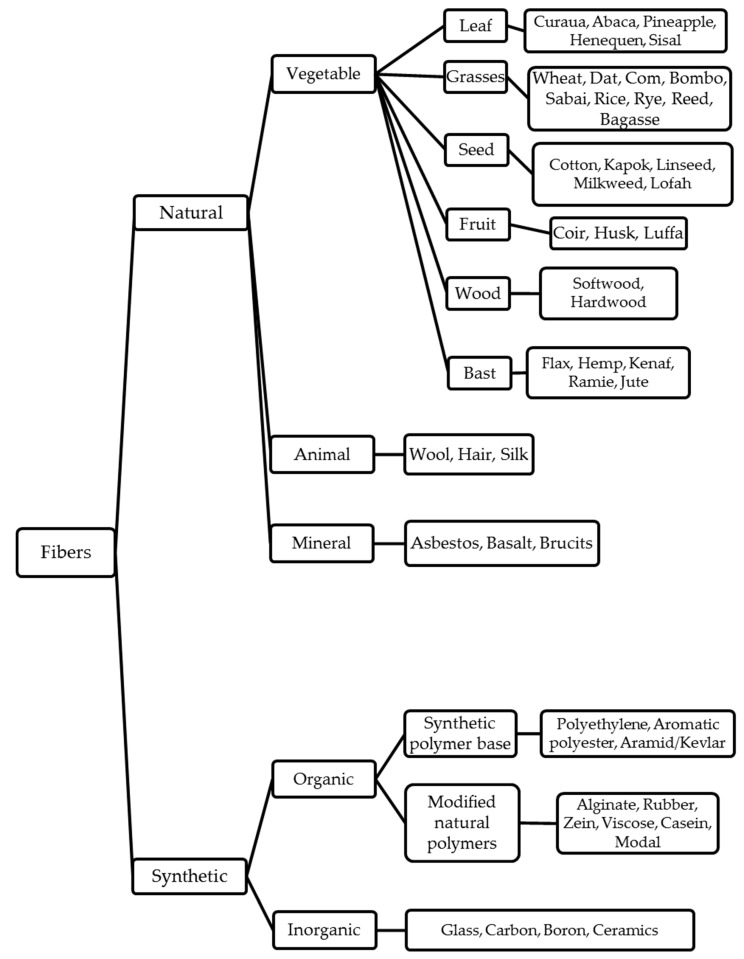
Classification of natural and synthetic fibers. Adapted from [38].

Fibers’ function as a discontinuous reinforcement phase in fiber-reinforced composites is modifying properties to obtain desired enhancement in the composite structure’s strength or/and stiffness. They can be subdivided into synthetic or natural fibers according to their origin, as illustrated in Figure 4 [37,38]. 

#### 3.1.1. Natural Fibers

**Figure 5:**
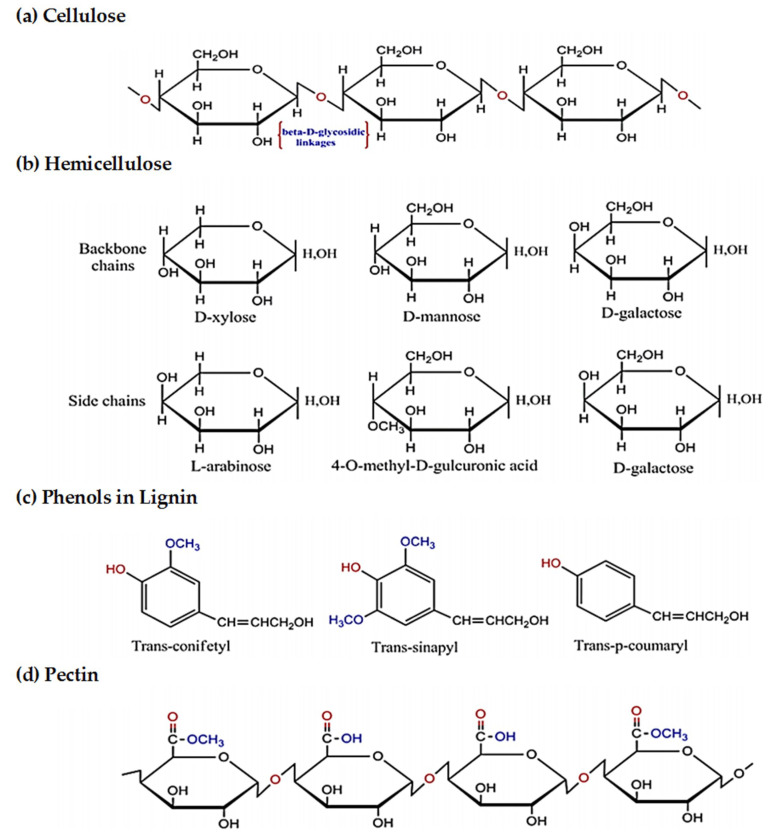
Chemical structure of the major constituents in the natural fibers: (**a**) cellulose; (**b**) hemicellulose; (**c**) lignin; (**d**) pectin. Adapted from [38].

Natural fibers can be extracted from different renewable resources of animals, vegetable plants, and minerals. The major constituents of natural fibers are cellulose, hemicellulose, lignin, and pectin, and their general chemical structures are illustrated in Figure 5 [39]. 

Cellulose, which is the most abundant organic polymer on earth, includes plenty of β-D-glucose (C_6_H_11_O_5_) units linked by glycoside linkages at the C_1_ and C_4_ carbon positions (Figure 5a) [40,41]. Cellulose, which is strong and durable, exists in both microcrystalline and amorphous structures with regions of a high order and low order, respectively [1]. It is resistant to strong alkali and oxidizing agents. Still, it is susceptible to hydrolysis with acid to water-soluble sugars and also is degradable when exposed to chemical and solution treatments [13]. 

Hemicellulose is the second-largest biomolecules, which includes polysaccharide units comprised of a highly branched polymer of carbon ring sugars (Figure 5b). It has a lower degree of polymerization and much shorter chains in comparison to cellulose. Moreover, it is hydrophilic and soluble in alkali solutions [26,42]. It can bond to the cellulose and lignin components in the cell wall to increase fibers’ rigidity and flexibility [43].

Lignin is a complex nanocrystalline molecule with hydroxyl, methoxyl, and carbonyl functional groups; however, lignin’s exact chemical nature remains unknown (Figure 5c). Lignin is formed within a plant cell wall composed of aromatic units and is amorphous and insoluble in most solvents. This biochemical polymer is a high molecular-weight phenolic compound, generally resistant to microbial degradation, and cannot be broken down to a monomeric unit [1,2,44,45]. When the hydrophobic lignin acts as a compatibilizer between hydrophilic cellulose and hemicellulose, it can improve the plant’s stiffness [46,47].

Pectin includes a complex group of heteropolysaccharides called galacturonoglycans with different methyl ester content. The simplest pectin, as presented in Figure 5d, is homogalacturonan (HG) and an unbranched polymer of α-(1–4) linked D-galacturonic acid [48]. Pectin indicates the luster and touch of fibers and acts as a binder of fibers into the bundles [45]. 

Wax makes mainly the outer part of the natural fibers and consists of different types of alcohol, which are soluble in water and acids. Wax provides a soft touch, low friction that results in fibers moving [45,49]. This constitution also affects the wettability and adhesion between fiber and matrix [50].

Natural fibers’ chemical composition, which depends on plant species, age, climate, and soil conditions, is crucial in their performance and application. Each constitution can induce different types of susceptibility to the natural fibers, i.e., biological, chemical, mechanical, thermal, photochemical, and aqueous. For example, cellulose is a governing constitute to improve the strength and modulus of fibers. Hemicellulose is in charge of biological and thermal degradation besides high water absorption (2.6 times higher than lignin); however, lignin mainly controls UV and fire degradation [43,51,52,53]. Another critical parameter of natural fibers that highly affect their mechanical properties is microfibrils’ orientation to the cell axis, determining the fibers’ stiffness [54]. Table 1 shows the chemical composition and structural parameters of common natural fibers.

The relatively good specific mechanical and physical properties of natural fibers are due to their low density and particular microstructure. Table 2 compares the physical and mechanical properties of some natural and synthetic fibers. Furthermore, the natural fibers’ pros and cons with respect to the synthetic fibers are listed in Table 3. Since the pros and cons depending on the application of fibers, it is worth mentioning that Table 3 was written to utilize fibers in biocomposite with environmental, economic, recycling, and eco-efficiency concerns. In this regard, for example, breaking down the fibers with micro-organisms to CO_2_, methane, and biomass (biodegradability) would be an advantage for environmental concerns. 

#### 3.1.2. Engineered Fibers

Engineered or synthetic fibers can be either organic or inorganic. The organic fibers are commonly divided into modified natural and synthetic polymers (Figure 4). The inorganic fibers are mostly derived from petroleum-based by-products and a combination of different chemicals. Glass, carbon, and aramid fibers are the most frequently used synthetic fibers in composites in varied areas of aerospace, defense, construction, sports, naval, etc. One of these fibers’ benefits over natural ones is that they are independent of growing conditions and can be manufactured with distinct functionalities needed for the proposed application [29,71]. Synthetic fibers are well known for their superior mechanical and thermal properties as compared to natural fibers. For example, carbon fibers with a wide range of aspect ratio, low thermal expansion, high stiffness, tensile strength, chemical resistance, and temperature tolerance are utilized as multifunctional fillers to improve polymers properties [72]. Glass fibers with low cost, high tensile strength, high chemical resistance, and excellent insulating properties are employed to manufacture the largest group of composite materials that is glass fiber-reinforced polymer (GFRP) composites. Glass fibers with varied forms such as long longitudinal, woven mat, chopped strand fiber, and chopped strand mat are incorporated into matrix to enhance mechanical and tribological properties of polymer composites [73]. Aramid fibers are mainly employed in high temperatures and high resistance applications such as manufacturing body parts in the aerospace and automobile industry, ballistic accessories in the military, boat hulls, heat-resistant helmets, etc. Their specific properties include high abrasive resistance, good fabric integrity at elevated temperatures, no melting point, high degradation temperature (starting from 500 °C), and low flammability [29,73].

### 3.2. Matrix Selection

A matrix in the composites provides the overall durability, including surface appearance, shape, and environmental tolerance. Another function of the matrix is to efficiently bind the fibers to transfer load between them [74]. These matrices depending on the processing technique and type of bonding present in them can be classified into thermoplastic and thermosets. Major polymers used as a matrix for composites are presented in Table 4.

Both thermoplastics and thermosets polymers have certain advantages and disadvantages as matrix materials in polymer matrix composites (PMC). The primary difference between thermoset and thermoplastic polymers is their behavior in exposure to heat. Thermoplastics can be reheated, remolded, and reprocessed into a new shape without any chemical changes, while thermosets that strengthen after heating cannot be remolded or dissolved in a solvent [74,75]. This behavior is attributed to the polymer chains’ interaction with each other. This interaction in thermosets is strong covalent bonds and chains have a highly cross-linked structure [76]. Therefore, thermoset resins such as epoxy, polyester, and polyamide have excellent dimensional stability and resistance against high temperatures without losing their structural integrity. Moreover, they have limited water absorption, high modulus, high strength, and good chemical resistance. The major drawback of thermosets is that recycling and reprocessing are virtually impossible after the initial forming [77,78]. On the other hand, thermoplastics that also have non-covalent bonds (i.e., relatively weak Van der Waals forces between long-chain molecules) are often chosen over thermosets due to their reversible changes, recyclability, moldability into various shapes, relatively low processing costs, and easy manufacturing with high volume and precision [79].

**Figure 6:**
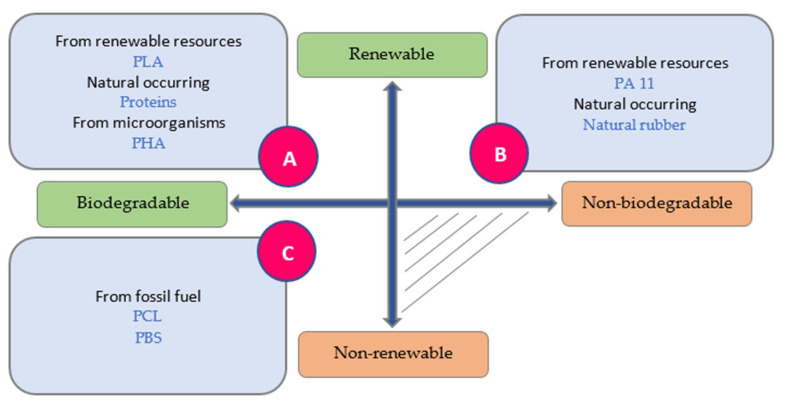
Biopolymer matrices classifications [©MB, JA, MAM_UC3M2020].

Resins or matrices in the concept of biopolymer can be defined by two criteria (Figure 6): (1) biodegradability of the polymer, (2) source of raw materials [80]. The first group (A) of resins are biodegradable biopolymers made from renewable raw materials (bio-based). The second group (B) of resins are non-biodegradable biopolymers made from renewable raw materials (bio-based). The last group (C) of resins are biodegradable biopolymers made from fossil fuels. The biodegradable bio-based resins (type A) are divided into three groups: (1) synthetic polymers from renewable resources, e.g., PLA; (2) biopolymers generated by micro-organisms, e.g., PHA; (3) natural occurring biopolymers, e.g., starch or proteins. The non-biodegradable bio-based resins (type B) that can be produced from biomass or renewable resources are divided into two groups: (1) synthetic polymers from renewable resources such as specific PAs from castor oil (PA 11); (2) natural occurring biopolymers, e.g., natural rubber or amber. The biodegradable resins of type C, such as polybutylene succinate (PBS) and polycaprolactone (PCL), are produced from fossil fuel such as synthetic aliphatic polyesters made from crude oil or natural gas [59,80,81]. Accordingly, not all the bio-based polymers are biodegradable, and not all the biodegradable polymers are bio-based, while some belong to both, such as PHA. Based on the above categories, the biopolyethylene derived from sugarcane, namely “green polyethylene,” developed recently, is classified in type B of novel biopolymers [82].

#### 3.2.1. Bio-Based Thermoplastics

The bio-based thermoplastics with renewable resources have attracted significant attention as an alternative to oil-based thermoplastics in recent years. In this section, some of the most critical bio-based thermoplastics are explained.

- Polylactic acid

**Figure 7:**
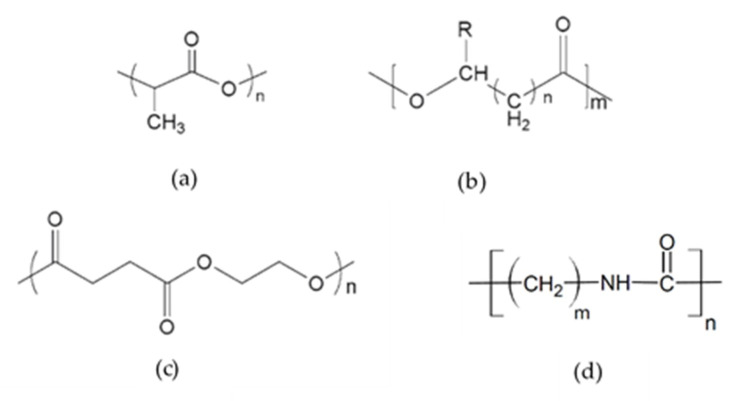
Chemical structure of (**a**) Polylactic acid (PLA), (**b**) Polyhydroxyalkanoate (PHA), (**c**) Poly butylene succinate (PBS), (**d**) Polyamide (PA). Adapted from [120,121,122,123].

Polylactic acid (PLA) is the most extensively explored and utilized biodegradable thermoplastic material produced from plant-derived carbohydrates such as glucose and starch, which are achieved from corn, potatoes, beets and cane, and so forth [83,84]. PLA with backbone formula (C_3_H_4_O_2_)_n_ (Figure 7a) is a hydrophobic polyester with a basic monomer of lactic acid. This monomer can be polymerized to PLA by direct polycondensation, ring-opening polymerization, solid-state polymerization, and azeotropic condensation polymerization [85,86]. PLA polymer can be amorphous, semi-crystalline, and highly crystalline polymer depending on the stereoisomer composition. PLA’s mechanical properties depend on various parameters, e.g., component isomers, processing temperature, annealing time, and molecular weight (Mw). Generally, it has high strength and moderate barrier properties. Simultaneously, it is relatively less thermally stable, decomposing below 230 °C and has a poor toughness with a low deformation at the break. For example, semicrystalline PLA, which is preferred over the amorphous PLA from the mechanical properties point of view, has an approximate tensile modulus of 3 GPa, a tensile strength of 50–70 MPa, and an elongation at break of about 4% [86,87,88].

- Polyhydroxyalkanoates

Polyhydroxyalkanoate (PHA) is a family of water-insoluble, biodegradable, and biocompatible natural polyesters that are biosynthesized from micro-organisms, modified plants, or through chemical reactions [89,90]. This thermoplastic polymer covers a broad range of mechanical properties, from hard and brittle poly(3-hydroxybutyrate) (PHB) to soft and elastomeric poly(3-hydroxyoctanoate) (PHO) [91]. PHB homopolymer (Figure 7b) is the main variant of the PHA family that is highly crystalline (50–70%), relatively hydrophobic, and an excellent gas barrier. Moreover, it has an elasticity modulus of 3 GPa, a tensile strength at break of 25 MPa, and has similar physical properties to conventional plastics such as polypropylene [92,93,94]. Due to the high melting temperature of PHB (about 173–180 °C), which is very close to its thermal decomposition temperature, it is hard to process it. Another drawback of PHB is an extreme brittleness due to the large spherulite formation and secondary crystallization [95]. The first attempt to overcome the mentioned disadvantages was the copolymerization of 3-hydroxybutyrate with other monomers to reduce the melting point and confer less stiffness [96]. Thus, various copolymers have been biosynthesized, such as poly(hydroxybutyrate-co hydroxyvalerate) (PHBV), poly(hydroxybutyrate-co-3-hydroxyhexanoate) (PHBHx), poly(hydroxybutyrate-co-hydroxyoctanoate) (PHBO), and poly(hydroxybutyrate cohydroxyoctadecanoate) (PHBOd) [95,97]. Furthermore, the reinforcing of PHAs with natural fibers could be another solution for brittleness and low-impact resistance [45].

- Polybutylene succinate

Polybutylene succinate (PBS) is one of the most favorable biodegradable polyesters, which is chemically synthesized through polycondensation of 1,4-butanediol and succinic acid or its anhydride in the presence of a catalyst [98,99]. This thermoplastic is a promising biopolymer owing to its mechanical properties and other beneficial properties, such as biodegradability, high crystallinity, melt processability, and thermal and chemical resistance. PBS consists of polymerized butylene units succinate with backbone formula (C_8_H_12_O_4_)_n_ (Figure 7c). It is fully decomposed into biomass, CO_2_, and H_2_O like PLA [100,101]. Although PBS has many exciting properties, its softness, high electrical resistivity, and low gas barrier limit this polymer’s practical applications. A diverse array of matrix polymers has been used to modify the PBS properties, such as PLA, poly(ε-caprolactone) and poly(hydroxybutyrate). Furthermore, adding reinforced fillers such as carbon nano tube (CNT), carbon black, layered silicate, and organoclay would be another method to improve PBS properties [102,103,104,105].

- Polyamide

Polyamide (PA) is a significant thermoplastic consisting of amide linkage (–NH–C=O) (Figure 7d). Polyamides that are crystalline polymers and commonly known as nylon can be classified into many categories depending on monomers’ arrangement and chemical nature. Aromatic, cycloaliphatic, and aliphatic polyamides are the most important ones [106,107]. Biopolyamides include both bio-based and biodegradable fossil fuel-based polyamides [80]. One of the most imperative bio-based polyamides is PA11, which is produced from 11-aminodecanoic acid derived from castor oil [108]. PA11 is a commercial aliphatic polyamide used in various fields, including automotive, food packaging, and offshore applications, thanks to its relatively good oil and saltwater resistance, biocompatibility, and lower hydrophilicity than PA 6 and 66 [109,110]. PA11 is a semi-crystalline polymer that is rigid at room temperature with a glass transition temperature (Tg) of 43 °C [111]. Various efforts have been reported to improve the properties of PA11 that most of them have focused on the blending of PA11 with other polymers, especially bio-based ones like PLA [109,112,113], PHA [114,115] and PBS [108,116] and also the incorporation of PA11 with inorganic fillers [117,118,119].

#### 3.2.2. Bio-Based Thermosets

The raw material for bio-based thermosets can be in the form of oil or other liquids extracted from diverse plants and animals. For example, verity thermoset resins are oil derived from fish, corn, soybean, cashew nutshell, linseed, etc. [124]. Thermosetting bioresins can be divided into epoxy, phenolic, polyurethane, polyester, and other resins. Table 5 presents a few thermoset bioresins available commercially on a large scale for industrial applications, and the bio-based content can be varied from 18% to 50–90% in them.

- Epoxy resins

Epoxy resins are one of the most functional thermosetting polymers often synthesized by the reaction of polyols, polyphenols, or other active hydrogen compounds with epichlorohydrin [127]. Thermosetting epoxy resins can provide various properties depending on the curing agents and proportions, curing cycles, and additives added during their production [128]. Tensile strength for epoxies is in the range of 90–120 MPa, and the tensile modulus ranging from 3100 to 3800 MPa [129]. According to their Tg, which varies from 150 to 220 °C (for uncured epoxies), they are the first or second common resin systems in aerospace. Apart from the properties mentioned above, epoxy resins’ main drawbacks are their brittleness, moisture sensitivity, and recycling difficulty [129,130]. Since most of the conventional epoxy resins are strongly dependent on fossil sources and also their raw materials are hazardous for human health and the environment (such as diglycidyl ether bisphenol A (DGEBA)), a significant amount of research is dedicated to finding more environmentally friendly epoxies [131,132]. In this regard, after high demand for green industries, thermosetting bio-based epoxies such as natural oil-based epoxies, isosorbide-based epoxies, furan-based epoxy systems, phenolic and polyphenolic epoxies, as well as epoxy lignin derivatives, have been introduced in recent years [133].

- Phenolic resins

Phenolic resins are one of the favorable thermoset polymers that retain their industrial and commercial interest a century after their introduction due to their appropriate characteristics, such as superior mechanical strength, heat resistance, infusibility, flame retardancy, dimensional stability, as well as high resistance against numerous solvents, acids, and water [134]. They have been widely utilized in the manufacture of wood products, molded plastics, and aerospace components. Phenolic resins are prepared by the reaction of formaldehyde (F) with phenol (P). They can be synthesized in both acidic and alkaline environments depending on the F:P molar ratio, either acid-catalyzed resins (called Novolacs) (0.75 < F:P ratio < 0.85) or alkaline-catalyzed resins (called Resoles) (F:P ratio > 1.0) [135]. Recently, many attempts have been made to replace petroleum-based phenolic resins with bio-based phenolic products from biomass feedstock by liquefaction and pyrolysis process [136,137]. The U.S. National Renewable Energy Laboratory (NREL) has reported that it is possible to produce phenolic resins from wood rather than fossil fuels with about half the cost [138]. The principal chemical compounds of biomass are cellulose, hemicellulose, and lignin. Forestry residues/wastes that contain 40–45% cellulose, 15–35% hemicellulose, and 20–35% lignin would be valuable resources for bio-based phenolic resins [139,140].

- Polyester resins

Polyester resins are the most economical resin systems utilized in engineering applications, particularly in the maritime and automotive industries [141,142]. Polyester is a viscous liquid resin produced by a condensation reaction between a glycol and an unsaturated dibasic acid. It is consists of a double bond between its carbon atoms (C=C) and is recognized by clear pale yellow color [143]. Unsaturated polyester resins (UPRs), which are vital cross-linkable polymeric materials, are extensively used as a matrix for fiber-reinforced composites in a broad range of engineering applications such as construction, automotive, marine, electrical, decorative, and aerospace industry [144,145]. They have the right balance of mechanical, thermal, electrical, and chemical resistant properties, easy processability, low density, and low cost [146,147]. Plant oils are the primary choice between all biomass-derived feedstocks to prepare UPRs with renewable resources, owing to their abundance, low toxicity, biodegradability, and triglyceride structures appropriate for further chemical modification [148,149]. There is a growing interest in incorporating natural oils or their derivatives into UPR, aiming to fabricate novel structural bioplastics. For example, Mehta et al. [150] used methyl ester of soybean oil and epoxidized methyl linseedate (EML) to produce biocomposites containing the modified UPR. Das et al. [151] developed novel biomaterials through blending UPR with tung oil with improved impact strength.

## 4. Mechanical Properties of Hybrid Biocomposites

Mechanical properties of hybrid biocomposites may depend on various features such as reinforcements and matrix mechanical properties, reinforcements dispersion and distribution, reinforcements volume fractions and aspect ratio, interfacial adhesion between polymer and reinforcements, loading and test conditions, fibers dimension and orientation, as well as surface modification [16,152,153,154,155,156]. In the following sections, some of the mechanical properties of recently developed hybrid biocomposites are reviewed.

### 4.1. Strength (Tensile, Flexural, Impact)

Mechanical testing, such as tensile, flexural, and impact tests, is the most common investigated hybrid biocomposites feature. The tensile strength of fiber-reinforced biocomposites generally increases with fiber content, up to optimum value, then will drop. This is due to the much higher strength and stiffness values of fibers than polymeric matrices [157,158,159]. When the natural fibers are introduced to the hydrophobic polymer matrices, their hydrophilic nature contrasts with matrices that induce high water absorption and subsequently result in low tensile strength of biocomposites. The surface treatment is a crucial solution to modify the fiber hydrophobicity and interfacial interaction of fiber/matrix, leading to improved tensile properties [160,161,162]. The longitudinal tensile modulus follows the linear rule of mixtures in the hybrid biocomposites, as many researchers have reported [35,75,163,164,165]. 

**Figure 8:**
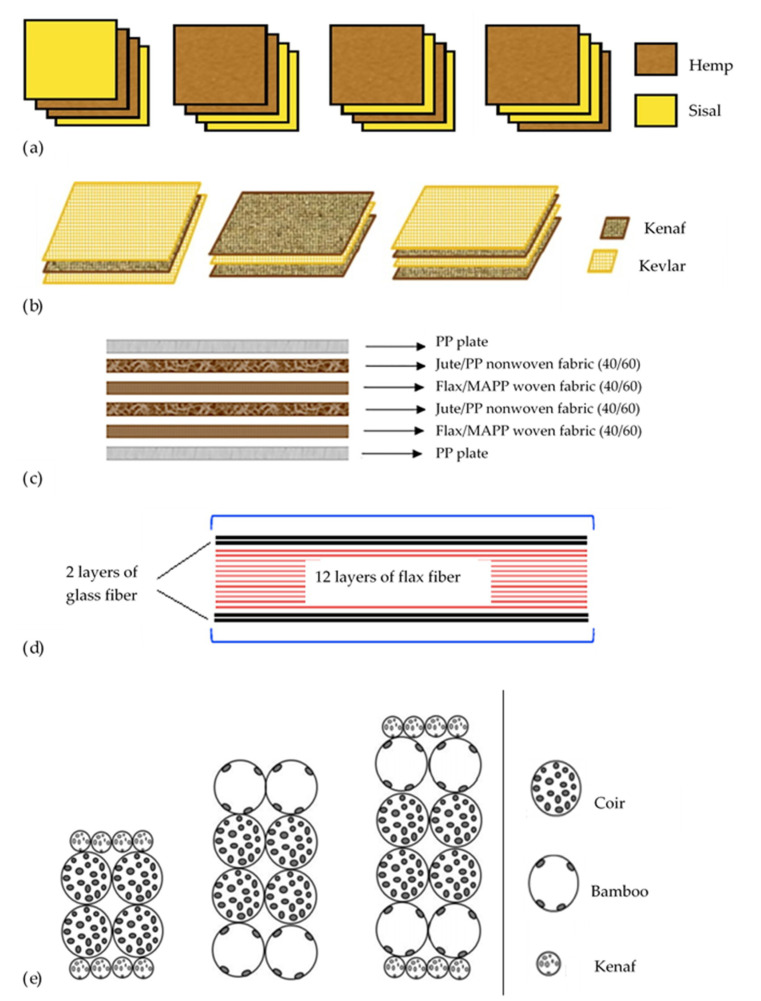
Different stacking configurations of some laminated hybrid biocomposites: (**a**) Hemp/sisal/epoxy. Adapted from [177], (**b**) Kenaf/kevlar/epoxy. Adapted from [178], (**c**) Jute/flax/ Polypropylene (PP). Adapted from [179], (**d**) Glass/flax/epoxy. Adapted from [180], (**e**) Coir/kenaf/epoxy, bamboo/coir/epoxy and kenaf/bamboo/coir/epoxy. Adapted from [181].

Impact resistance is another vital property of hybrid biocomposites, which is strongly related to toughness. It can be identified by energy absorption during a penetration impact, residual properties after impact, and damaged area after a nonpenetration impact. Moreover, dispersion and positioning of layers in hybrid biocomposites layup are known to be essential parameters for impact [166]. Laminated composites are the most common manufactured configuration among the various composites’ structures. Figure 8 presented a few samples of the recent hybrid biocomposites with laminated design and different stacking sequences of layers. According to these configurations and other reported results [167,168,169,170,171,172], positioning of fibers with the highest energy-absorption on the outside layer rather than inside allows the hybrid composite to absorb more energy and achieve higher penetration impact resistance consequently. Furthermore, a higher degree of dispersion demonstrated better penetration impact resistance due to the smaller delaminated area and higher interfaces between distinct layers that absorb more energy [173,174,175]. Impact performance is also sensitive to interfacial adhesion between fiber and matrix [53]. The low adhesion in natural fiber-reinforced composites (NFRCs) and the load transfer between fiber and matrix would be improved by hybridization with synthetic fibers. In contrast, even synthetic reinforcements such as carbon fibers need to increase their surface energy by surface modifications to ensure the proper adhesion with matrices [176].

The flexural properties of hybrid composites, which reflect the laminate stiffness and dimensional constancy, are more difficult to understand than tensile properties [81]. The flexural strength and modulus are strongly dependent on the layup, as the longitudinal stress increases by moving away from the neutral line. Besides, changing the ply angles, material type, and stacking sequences of fibers can alter the flexural properties [166,182].

**Figure 9:**
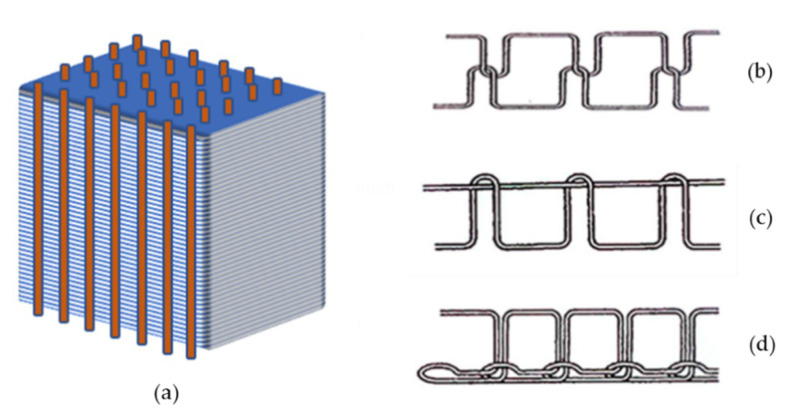
Different reinforcement stitching techniques: (**a**) Z-pinning [©MB, JA, MAM_UC3M2020], (**b**) lockstitch, (**c**) modified lockstitch, (**d**) chain stitch. Adapted from [200].

**Figure 10:**
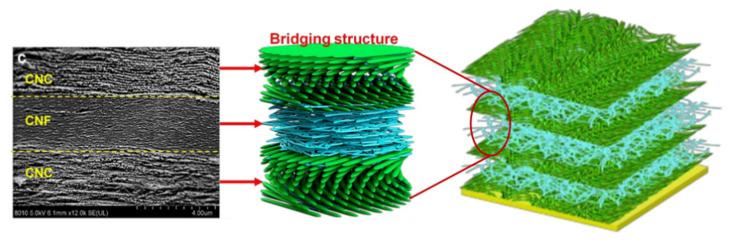
Schematic of chiral nematic cellulose nanofiber/random cellulose nanofiber (CNC–CNF) biocomposite with an alternating sequence of layers and corresponding SEM image of layers. Adapted from [199].

As mentioned earlier, the impact properties of polymeric materials depend essentially on the toughness of them. The material’s toughness can be referred to as the material’s capability to absorb the dynamic impact energy [183]. Natural fiber-based composites have desired interfacial properties and can dissipate a large amount of dynamic impact energy through the breakage and pull out the fibers. Moreover, hybridization of natural fiber composites with synthetic fibers is an effective technique to improve mechanical properties, especially the toughness [184]. Glass fibers—the most common synthetic fibers for hybridization of natural fibers—have high mechanical strength and good mechanical interlocking with hydrophobic matrices [185]. The most effective routes for hybridization of glass and natural fibers are (i) effective dispersion of short glass fibers with small loading into the bulk of short natural fibers, and (ii) laminated hybrid composite with glass fibers plies as an external skin and natural fibers as core laminates [186]. For example, in the glass/hemp hybrid composite, higher impact damage tolerance and toughness can be obtained using 11 vol % of glass fibers to reinforce the skin of hemp fibers composites due to superior stiffness and bending strength of glass fibers [187]. Another example is employing the small loading of glass fibers (up to 8.6 wt %) in alkali-treated sisal fibers and pineapple leaf fibers (PALF) to improve the toughness and impact strength (up to 87%) of hybrid composites [188]. Furthermore, it has been reported that the delamination fracture toughness of the glass/silk hybrid composite can be enhanced by placing the silk fabrics in the middle of the laminate composite to hinder the propagation of crack and control the crack delamination through the bending process [189]. The toughness of composites also depends on the fabrication method. Among the various techniques to improve interlaminar fracture toughness, through-the-thickness stitching is a promising technique using reinforcements at out of plane directions. In this technique, the composite can be fabricated by (i) reinforcement of un-cured pre-preg (pre-impregnated) laminates in the direction of a thickness (Z-pinning technique) (Figure 9a), and (ii) employment of dry fabric preforms that already includes the through-thickness reinforcement before the resin infusion (stitching). Modified lockstitch, lockstitch, and chain stitch are commonly used stitching techniques (Figure 9b–d) [190,191,192,193]. As for the weave preform, the best structural arrangement is the twill weave fabric due to its having the highest impact strength, which results in higher required force to pull out or break the fibers [194]. Moreover, incorporating nano and micro fillers has significant effects on the fracture properties of multi-scale composites. Nano and micro fillers contribute to toughening mechanisms by bridging micro-cracks and slowing down the crack initiation and crack growth. The average weight fraction required to enhance the composites’ toughness and mechanical properties is in the range of 0.1–10 wt % and 10–50 wt % for the nano and micro-fillers, respectively [195,196,197,198]. Recently, Zhang et al. [199] reported robust laminated biocomposites with improved mechanical strength and toughness. Figure 10 shows how a bridging structure is formed between alternating layers of chiral nematic cellulose nanocrystal (CNC) and random cellulose nanofiber (CNF) phase. This configuration would be useful when there are ductile and brittle phases in the composite. The more ductile material (CNF) has a mechanical buffering performance with the alternating sequence of layers. It prevents the initiation and propagation of cracks within the brittle material (CNC). Moreover, the ductile CNF layers provide strong hydrogen bonding networks with brittle CNC layers that optimize load transferring between brittle and ductile layers and improve strength and toughness. This manufactured biocomposite increases damage-tolerant property for soft-robotics and colorimetric sensors. Table 6 summarized the recent signs of progress on the mechanical properties of hybrid biocomposites.

### 4.2. Water Absorption

All green composites may absorb water in a humid atmosphere or when immersed in water. Natural fibers with a hydrophilic nature due to hydroxyl (–OH) and other polar groups in their different constituents such as cellulose and hemicellulose are interested in absorbing water. This phenomenon leads to swelling of the fiber, degradation of a fiber-matrix interface, plasticizing effect, expansion of the gap between fiber bundles that reduces the load-transfer efficiency, and results in depletion of biocomposite performance and reduction of mechanical properties [239,240,241]. Accordingly, an inevitable step before using biocomposites in each application is analyzing the water absorption of the developed biocomposite. Water absorption in composites can be affected by fiber volume fraction, a viscosity of matrix, voids, humidity, and temperature [242]. Three various mechanisms govern water diffusion in green composites. The first one is the diffusion of water molecules inside the micro gaps of polymer chains. The next one is the capillary movement of water into the flaws and holes at the fiber-matrix interfaces. The last one is the initiation of microcracks in the matrix resulting from swelling of fibers or laminates [243,244,245]. Fibers’ surface treatments as a solution approach to reduce the water uptake have attracted several researchers. Treatment techniques of reinforcement fibers can be generally classified into chemical and physical methods. The fiber’s structural and surface properties are modified by employing physical modification methods, resulting in changing the mechanical bonding between the fiber matrix. Stretching, calendaring, and electric discharge with corona and cold plasma are some examples of physical modification methods [246,247,248,249,250,251]. Thermal treatment is another physical approach to modify water absorption property in biocomposites. It causes the fibers’ moisture loss, increasing interfacial adhesion, and fiber stiffness due to the increased crystallinity [252,253]. It is noteworthy that the increase of fiber-matrix adhesion is a prerequisite for high strength and leads to lower water uptake [253]. Aging that is wet/dry cycling treatment and induces shrinkage and reduces the water retention values is an effective thermal treatment in this matter [16,254]. On the other hand, chemical modification techniques adjust the hydrophilic hydroxyl groups from the fiber surface based on reagent functional groups, reacting with functional groups in the fibers, and altering their compositions [44]. Various chemical treatments such as alkali, benzoylation, mercerization, silane, acetylation, isocyanate, acrylation, permanganate, peroxide treatment with multiple coupling agents, and bio-based coatings have been applied on fiber-reinforced biocomposites to reduce water absorption, improve fiber-matrix adhesion, and consequently enhance mechanical performance [57,241,253,255,256,257,258,259]. 

Another reported approach to increase the water-resistance of hybrid biocomposites is the integration of nanofillers. Three types of nanofillers have been used in researches to enhance the performance of composites (i) nanotubes, (ii) nanoparticles, and (iii) nanolayers. Nanofillers with a high aspect ratio can be made of metals, metal oxides, polymers, and carbon [260]. Ramesh et al. [261,262] worked on the hybridization of PLA/treated kenaf and PLA/treated aloevera biocomposite with montmorillonite (MMT) nanoclay. In both cases, adding the MMT nanoclay successfully increased the hybrid biocomposites performance, especially water resistance. This phenomenon is referred to as the barrier function of nanoclays, limiting the flow of water into biocomposite and reducing water absorption. Nanoclay particles, which are classified under silicate nanomaterials, are one of the most commonly utilized nanofillers. They can be added in composites with various forms, such as nanoclay platelets, calcinated nanoclay, and montmorillonite. Anbukarasi et al. [263] investigated the effect of SiO_2_ nanospheres fillers on the water absorption behavior of luffa-coir/epoxy hybrid composites. They concluded that the water absorption of the hybrid composite was significantly reduced by adding SiO_2_ nanospheres. These nanospheres fillers compensate for the fibers’ hydrophilic effect by reducing the number of free hydroxyl groups of luffa and coir fibers. SiO_2_ nanoparticles have high strength and high specific surface area, which improves the mechanical properties of composites and enhance the interfacial adhesion between fibers and matrix [264]. Hasan et al. [265] evaluated the effects of halloysite nanotube (HNT) on epoxy/basalt hybrid biocomposites’ durability. They reported adding two wt % HNTs provides better interfacial bonding between fibers/matrix and decreases water absorption. Better water resistance is mostly due to the 1D morphology of the HNTs and their high aspect ratio, which restricts the diffusion of water molecules by means of a tortuous path.

Water adsorption would also be a desired property in applications in which high water intake is more favorable than mechanical properties such as tissue engineering, biomedical, and biotechnology. Superabsorbent polymer composites (SAPCs) or hydrogels are among the most well-known materials that require high water absorption capacity more than their dry mass [266]. Many researchers have recently reported novel synthetized biobased SAPCs [267,268,269,270,271] and hybrid hydrogel composites [267,268,269,270]. Only a few studies have been recently reported hybridization of hydrogels with fibers [271,272,273], and Table 7 is excluded from them because adding fibers in those cases is to modify other properties of hydrogel rather than water absorption. Table 7 lists the recent findings on the water absorption behavior of hybrid biocomposites.

### 4.3. Flame Retardancy

One of the major drawbacks of biocomposites is their relatively poor resistance to burning. Most of the natural reinforcements and biopolymer (hemicellulose and lignin) are susceptible to fire and combustion. They undergo thermal decomposition at low temperatures (200–300 °C) [286,287]. Since natural reinforcements’ flammability could limit their application in the automotive, aerospace, and construction industries, it is vital to adjust materials with more flame-resistant without compromising their good mechanical properties [27]. Recently, there has been more research on hybrid materials based on synthetic-natural fibers to manufacture more environmentally friendly composites. Many researchers reported that by incorporating synthetic fibers into the natural ones, it might be possible to increase the thermal stability and flame resistance of biocomposites. Some of these recent studies on the flammability performance of hybrid biocomposites are summarized in Table 8. Furthermore, another attractive approach to reducing the flammability issue is the hybridization of flame-retardants (FRs) and natural fibers in the development process of biocomposites [288,289,290]. Many studies are currently attempting carbon-based materials as FRs because of their outstanding thermal, chemical, and mechanical properties accompanied by inherent resistance against combustion [291]. In this regard, different types of carbon-based nanofillers such as graphene [292,293], graphite [294,295,296], fullerene [297,298], graphene nanosheets (GNSs) [299,300], carbon nanotubes (CNTs) [301,302], multi-walled carbon nanotubes (MWCNTs) [294,303], graphene quantum dots (GQDs) [304,305], etc. have been reported to improve the flame retardancy of composites. Biochar is another carbon-rich material as FRs obtained by heating any biomass and renewable sources [306]. Biochar is a renewable alternative for inorganic carbon-based fillers (CNT, fullerene, etc.) [307]. The main function of carbon-based fillers is to form the protective char layer during the pyrolysis of polymeric substances to restrict the transfer of combustible gases and heat and therefore prevent further degradation of materials [291].

Generally, there are various techniques to achieve flame retarding polymeric composites [290]:

- Impregnation of fibers with a solution of the fire retardant
- Addition of microparticles or nanoparticles in matrix or reinforcement phase
- Direct incorporation of fire retardants
- Use of bio-based polymers that can potentially possess inherent fire retardancy
- Incorporation of the fire retardant into the adhesive system
- Mixing of fibers and fire retardant before adding an adhesive
- Chemical modification of matrix
- Surface treatment of fibers

FR materials which cause deceleration of combustion have some functions in composites, such as the reduction of produced heat to the stable level, the formation of a barrier between ambient oxygen (O_2_) and flammable polymers, release of bromine and chlorine atoms by the introduction of materials to polymers, and operation of pyrolysis process to limit the growing of flammable volatiles [308]. 

To determine the fire behavior of biocomposites, a variety of tests can be performed. For instance, in the limiting oxygen index (LOI) test, the minimum required amount of O_2_ to support flaming combustion of material can be measured [309]. Cone calorimetry is another attractive technique to evaluate the fire properties of materials. This test can give useful information in different stages regarding the time to ignition (TTI) (ignition stage), heat release rate (HRR) (fire developing stage), and total heat release (THR) (fully developed fire stage) [310]. Vertical burning test (UL-94) and micro-scale combustion calorimetry (MCC) are examples of other techniques to study the flame behavior of materials [291]. Table 8 lists the recent results on the flammability of hybrid biocomposites.

## 5. Conclusions

In this report, hybrid biocomposites have been reviewed, especially in mechanical properties, including tensile, flexural, and impact strength, water absorption, and flammability. In this regard, hybrid composites comprising natural-synthetic fibers, natural-natural fibers, and natural fiber hybrid with nanoparticles or fillers were investigated. Using multiple fibers and developing hybrid composites have received popularity due to the enhanced performance of manufactured products and the possibility of overwhelming the drawbacks of purely natural or synthetic fibers reinforced composites. The focus of this concept is to develop multifunctional materials and structures for advanced applications with improved properties. Researchers have applied various strategies in order to improve the mechanical properties of hybrid biocomposites. Adding a second filler in either micro or nano-size, change the weight percentage of natural and synthetic fibers, addition of fire retardants, change in layering stacking sequence, chemical or physical modification, and changing production techniques are some of the critical strategies. Despite significant challenges with compatibility and processing of hybrid materials, the aforementioned applied techniques showed remarkable improvements in strength, water absorption resistance, and flame retardancy.

To sum up, in the investigated studies in this review, hybridizations of natural and synthetic fibers with polymeric matrices as well as employing the optimized amount of substances, such as nanoclays, CNTs, silica, cement, and iron oxide particles, alumina, aluminum hydroxide, and graphite nanoplatelets effectively enhanced mechanical properties (e.g., flexural, tensile, and impact strength, flame, and water absorption resistance). High-performance glass and carbon fibers are still the most applicable synthetic fibers for hybridizing the natural fiber composites in recent years. Moreover, chemical treatments or modifications of fibers generally resulted in better mechanical properties, fire, and water resistance than untreated composites due to the improved fiber-matrix bonding after treatments. Among the processing methods, compression molding and hand lay-up are the most often used techniques for the hybrid composites with polymeric matrix materials. 

Undoubtedly, due to the rising environmental, economic, and application concerns, hybrid biocomposites are gaining significant attention in the future and provide a competitive market for numerous industrial applications. Consequently, this concept is still open, and further research on the possible ways to improve hybrid biocomposites’ properties has to be done. Modeling and simulation would be desirable facilitators for optimizing these materials properties and addressing the key required changes in production processes. 

## Figures and Tables

**Table 1:** Chemical composition of various natural fibers.

Fiber	Cellulose	Hemicellulose	Lignin	Pectin	Wax	Microfibrillar Angle	Ref.
	(%)	(%)	(%)	(%)	(%)	(°)	
Bast/Stem fiber							
Flax	64–81	14–20.6	2–5	0.9–2.3	1.7	5–10	[53,55,56,57,58,59]
Hemp	57–92	14–22.4	3.7–13	0.9	0.8	2–6.2	[53,54,55,58,59,60,61]
Jute	45–84	12–21	5–13	0.2	0.5	8	[57,58,59,60]
Kenaf	44–72	21–24	8–21	2–5	-	2–6.2	[38,53,55,61]
Ramie	68.6–76.2	5–16.7	0.6–1	1.9–2	0.3	7.5	[53,56,57,58,59,60]
Banana	60–65	6–19	5–10	3–5	-	11	[53,58,62]
Nettle	86	10	5.4	0.6	3.1–4	-	[38,60,61,63]
Leaf fiber							
Sisal	43–78	10–24	4–13	0.8–2	2	10–22	[53,55,57,58,60,62,64]
Curaua	70.7–73.6	4–9.9	7.5–13.1	-	-	-	[38,56,60,61]
Pineapple	80–83	15–20	4.6–12	2–4	4–7	8–15	[38,58,63,65]
Abaca	56–64	21–25	7–13	0.8–1	3	-	[56,58,60,62]
Henequen	60–77.6	4–8	8–13.1	-	0.5	-	[38,57,59,60]
Fruit/Seed fiber							
Cotton	82–96	2–6	0.5–1.6	0–7	0.6	20–30	[38,55,58,60]
Coir	36–46	0.15–0.3	41–45	3–4	-	30–49	[53,55,56,58,66]
Oil palm	65	0–29	19	-	4	46	[38,56,60,62]
Wood							
Hardwood	43–47	25–35	16–24	-	-	-	[38]
Softwood	40–44	25–29	25–31	-	-	-	[38,53]
Grass fiber							
Bagasse	32–55.2	16.8–21	19.9–25.3	10	-	-	[56,60,63]
Bamboo	26–43	15–30	21–31	-	-	-	[53,56,58,60]
Rice	28–57	33	8–19	8–38	-	-	[56]
Wheat	29–45	15–31	13–20	-	-	-	[53,56,60]

**Table 2:** Mechanical properties of some natural and synthetic fibers.

Fiber	Density (g/cm^3^)	Tensile Strength (MPa)	E-Modulus (GPa)	Elongation at Break (%)	Ref.
Bast fiber					
Flax	1.5	345–1100	27.6	0.2–3.2	[50,56,57,60]
Hemp	1.48	690	30–70	1.6–4	[54,56,57,60]
Jute	1.3–1.45	393–773	10.0–30.0	1.2–1.8	[50,56,57,60]
Kenaf	-	930	22.0–60.0	1.6	[50,56,60]
Ramie	1.5	400–938	44–128.0	1.2–3.8	[50,57,59,61]
Leaf fiber					
Sisal	1.5	468–640	9.4–22.0	3.0–7.0	[56,57,59,67]
Curaua	1.4	500–1150	9–11.8	3.7–7.5	[50,56,60]
Pineapple	1.5	413–1627	34.5–82.5	0.8–1.6	[38,50,62,63]
Abaca	1.5	400	12.0	3.0–10.0	[50,56,60,61]
Fruit/Seed fiber					
Cotton	1.5–1.6	287–800	5.5–12.6	7.0–8.0	[50,59,60]
Coir	1.2	131–175	4.0–6.0	15.0–40	[50,56,59,60]
Oil palm	0.7–1.55	248	3.2	25.0	[50,56,60,61]
Grass fiber					
Bagasse	1.25	290	11–17	-	[50,56,60,62]
Bamboo	0.6–1.1	140–441	11–17	-	[50,55,56,60]
Synthetic fiber					
Aramid	1.4	3000–3150	63.0–67.0	3.3–3.7	[50,57,59]
Carbon	1.7	4000	230–240	1.4–1.8	[50,57,59]
E-glass	2.5	2000–3500	70.0	2.5	[55,57,59,62]
S-glass	2.5	4570	86.0	2.8	[50,57,59,62]

**Table 3:** Natural fibers’ advantages and disadvantages. Adapted from [68,69,70].

Advantages	Disadvantages
Biodegradability	High water absorption
Renewability	Limited processing temperature
Low density	Dimensional instability
High filling levels and non-abrasive to the molding machinery	Poor fire resistance
Easily colored	Lower strength and lower thermal resistance
Non-abrasive to processing equipment	Anisotropic behavior
Good thermal and acoustic insulating properties	Sensitive to UV, microbial and fungus attach
Friendly processing atmosphere, non-harmful gases generation, reduced thermal and respiratory irritations	The volatility of properties and price based on the location
Zero fingerprint CO_2_	Incompatibility with most hydrophobic polymers
Low production energy	Poor fiber/matrix adhesion
Non-brittle fracture on impact	Lower strength, especially impact strength

**Table 4:** Major polymers used as a matrix for composites.

Polymers	
Thermoplastics	Thermosets
Polylactic acid (PLA)	Epoxy
Poly butylene succinate (PBS)	Phenolic
Polyhydroxyalkanoate (PHA)	Polyester
Polyamide (PA)	Polyurethane (PU)
Polyethylene (PE)	Vinyl ester
Polycarbonate (PC)	Silicone
Polyvinyl chloride (PVC)	Melamine
Polystyrene (PS)	-
Polypropylene (PP)	-
Polyurethane (PU)	-

**Table 5:** Examples of few commercially available bio-based thermosets.

Resin Type	Resin Base	Trade Name	Ref.
Unsaturated polyester	Soybean oil	Ashland’s Envirez	[124]
Acrylated epoxidized soybean oil (AESO)	Soybean oil	Ebecryl 860	[125]
Epoxidized soybean oil	Soybean oil	Vikoflex 7170	[126]
Epoxidized pine and vegetable oils	Pine and vegetable oils	Super Sap 100 Epoxy	[126]
Epoxidized linseed oil	Linseed oil	Vikoflex 7190	[126]
Acrylate functional resin	Soya oil	Cognis Tribest	[125]
Natural phenols	Distilled from forest industry waste stream	Amroy Europe Oy EpoBioX^TM^	[124]
Furfuryl alcohol-based resins	Biomass	TransFurans Chemicals bvba BioRez™ furfuryl resin	[124]
Alkyl phenolic oil	Cashew nut	CNSL	[126]

**Table 6:** Mechanical properties of recently developed hybrid biocomposites.

Matrix	Fibers	Manufacturing Process and Conditions	Mechanical Properties	Ref.
Vinyl ester/unsaturated polyester	Bagasse/henequen	Hand lay-up Alkali treatment (5% NaOH) Fiber length = 2 cm	TS ^1^ = 150 MPa FS ^2^ = 159 MPa IS ^3^ = 335 J/m	[201]
Epoxy resin (EpoxAmite 100) modified with multi-walled carbon nanotube (MWCNTs)	Flax/carbon (FLXC) Flax/glass (FLXG) Flax/Kevlar (FLXK)	Mechanical stirring and hand lay-up Dispersing agent: dimethyl ketone (2 propanone) with 1wt % CNT	Improvement of tensile properties with 1 wt % CNT TS _FLXC_ = 340.13 MPa TS _FLXK_ = 216.23 MPa TS _FLXG_ = 114.82 MPa	[154]
PLA	PALF/coir fiber (CF)	Hot press Fiber loading: 30 wt % CF:PALF = 1:1	Hybrid composite of C1P1 (15% CF 1 15% PALF) showed better mechanical properties respect to single fiber composites TM ^4^ = 4.75 GPa TS = 19.15 MPa FM ^5^ = 4.86 GPa FS = 33.04 MPa IS = 4.24 kJ/m^2^	[202]
Epoxy resin	Napier/carbon Napier/glass	Vacuum infusion Napier:carbon and Napier:glass = 30:0, 24:6, 18:12, 12:18, 6:24	Increase of carbon and glass vol fraction increased the flexural properties (max at 6:24% vol) FS: Napier/carbon biocomposites = 456.31 MPa Napier/glass biocomposites = 124.94 MPa FM: Napier/carbon biocomposites = 25.76 GPa Napier/glass biocomposites = 13.15 GPa	[203]
PLA	Kenaf/coir (KCCK) Bamboo/coir (BCCB) Kenaf/bamboo/coir (KBCCBK)	Hot press	TS of KBCCBK = 187 MPa (20 and 78% higher than BCCB and KCCK) FS of KBCCBK and BCCB = 199 MPa, 206 MPa (16% and 20% higher than KCCK) FM of KCCK = 15 GPa (70% higher than others)	[181]
EpoxAmite 100 with MWCNTs as a nanofiller	FLXC FLXG	Wet lay-up 1 wt % concentration of MWCNT	Better impact properties and higher compressive strength of FLXG compared to FLXC	[204]
Vinyl ester (VE)	PALF/glass	Automated spray up Vol. ratio of fibers = 50/50	TS (71.86 MPA) increased by 171% compared to PALF-VE composite FS (146.60 MPA) increased by 164.66% compared to PALF-VE composite	[205]
PLA-g-GMA	Agave fibers/nanoclay particles	Extrusion Compatibilizer: glycidyl methacrylate (GMA)	Using GMA caused an increment in TS and FS Nanoclay particles improved the tensile and flexural properties of the biocomposite	[206]
PLA	Alkali treated sisal and coir fibers (ASF and ACF)	Compression molding Sisal:coir ratio = 7:3	IS increased by 22.8% to PLA/ASF FS improved (92.6 MPa) Decline of TS	[207]
PLA	Treated Kenaf fiber (TKF)/montmorillonite clay (MMT)	Screw extruder and compression molding Alkali treatment (6% NaOH) Composition: 30TKF-1MMT-69PLA	FS and TS are improved by 46.41% and 5.87% than PLA/TKF	[208]
Epoxy polymer (RenLam M- 1 and Hardener HY 951)	Sisal/glass/portland cement particles	Hand lay-up Fiber-matrix mass fraction: 30/70 Stacking sequence: five layers of sisal/glass and glass/sisal	Increase of FS due to the cement microparticles and appropriate stacking sequence	[209]
PHB	Woven kenaf bast fiber (KBFw)/oil palm empty fruit bunches (EFB)	Lamination and compression molding Plasticizer: triethyl citrate (TEC). Arrangement: 11 layers (3KBF, 2EFB, 6PHB)	11-layer hybrid composite with improved mechanical properties can be an alternative for some woody products TS = 53.3 MPa TM = 5.4 GPa FS = 77.90 MPa FM = 7.3 GPa IS = 40.6 J/m	[210]
Epoxy resin	PALF/coir	Hand lay-up molding Application environment: natural soil Fiber/resin ratio = 40:60	Decrease of mechanical strength of hybrid composites in burial condition compared to the pure PALF-Epoxy composite	[211]
Polyester resin	Bamboo/PLF/coir	Hand lay-up followed by hot compression molding Fibers loading: 45%, 30% and 15% vol Bamboo:PLF:coir = 15:15:15, 10:10:10 and 5:5:5	Higher mechanical strength of hybrid composite with 45% vol fibers loading compared to the single fiber-reinforced composites TS: 136 MPa FS: 93 KN	[212]
Unsaturated polyester	Sugar palm yarn/glass	Sheet molding process and hot press Resin/fiber ratio = 70:40 wt % Sugar palm yarn:glass ratio = 50:50 wt %	TS, TM, FS, FM, and IS of the hybrid composites increased with increasing glass fiber loadings	[213]
polypropylene	PALF/banana	Compression molding Chemically treatment with 5% NaOH Fiber loading: 2, 5, 10 and 15 wt % PALF/banana ratio = 3:1, 1:1 and 1:3	The hybrid biocomposite with 5 wt % fibers loading and PALF/banana ratio of 3:1 exhibited the best set of mechanical properties	[214]
polyurethane	Sugar Palm/glass	Melt compounding and hot pressing molding process Chemical treatment: 6 wt % alkaline + 2 wt % silane solution	The TS, FS, and IS of a hybrid composite improved by 16%, 39%, and 18%, respectively, after the chemical treatment	[215]
Phenol formaldehyde	Areca fine (AF)/calotropis gigantea (CG)	Hand lay-up	Composite with 17.5 wt % CG and 17.5 wt % AF fiber had maximum tensile, flexural, and impact properties	[216]
Linear low-density polyethylene (LLDPE)	Sugarcane bagasse (SB)/eggshell (Es)	Compression molding Fibers treated with titanium (IV) isopropoxide and silane coupling agent	TM and FM of the composites with treated fibers were higher than untreated fibers Improvement of TM and FM with increasing of filler content up to 20/20 wt % The TS, FS, and IS tended to decrease with increasing SB/Es content	[217]
Phenol formaldehyde resin	Areca/sisal Areca/glass Areca/roselle	Hand lay-up Divinylbenzene cross-linking agent	Areca/sisal hybrid biocomposites presented the highest TS and TM than others FS and FM increased by hybridization of sisal, roselle, and glass fibers with areca	[218]
Thermoplastic SPS/agar (TPSA)	Sugar palm starch (SPS)	Hot press	The TS and FS slightly improved, but the IS reduced	[219]
Polyurethane foam	Roselle fiber (RF) with spherical silica (silica-A) and amorphous silica (silica-B)	Liquid molding	FM increased with increasing wt % of silica-A and silica-B TS increased with the increasing of silica-B and RF Adding silica-A up to 0.75 wt % also increased TS	[220]
Epoxy	Glass/Flax/Basalt (GFB) Flax/Hemp/Basalt (FHB) Glass/Hemp/Basalt (GHB)	Vacuum infusion process Stacking sequence: GFB: GFBBFG FHB: FHBBHF GHB: GHBBHG	Reinforcement volume: 21–23% Flexural performance: GFB > FHB > GHB	[221]
Polypropylene	Banana/Coir	Twin-screw extruder and injection moulding Fiber loadings (CF/BF/PP): 15/5/80, 10/10/80, and 5/15/80 wt %	Max strengths at Banana/Coir: 15/5 wt % TS: 31.3316 MPa TM: 760.29 MPa FS: 31.336 MPa FM: 762.326 MPa IS: 51.6 J/m	[222]
Epoxy	Banana/Jute	Hand lay-up Banana/jute ratio = 7:3	Better mechanical properties of the hybrid composite compared to mono composites TS: 85.91 MPa FS: 151.3 MPa FM: 1.23 GPa IS: 484.54 J/m	[223]
Epoxy	Banana/Kenaf	Hand lay-up Banana/kenaf ratio = 40:60, 45:55, 50:50, 55:45 and 60:40	Better mechanical properties with the highest kenaf %: TS: 58 MPa TM: 0.28 GPa FS: 24 MPa IS: 15.81 J	[224]
Epoxy	Sisal/Jute	Hand lay-up Jute/sisal ratio = 1:0, 1:3, 1:1 and 0:1	Fiber loading of 30 wt % Better mechanical properties of 1:1 hybrid composite TS: 102.08 MPa TM: 2.03 GPa FS: 361.9 MPa FM: 17.5 GPa IS: 30.1 KJ/m^2^	[225]
Polypropylene	Sisal/Glass (SG) Sisal/Carbon (SC)	Single extrusion machine and press consolidation SG and SC ratio = 25/75, 50/50, 75/25 wt %	Hybrid composite of 25/75 wt % for both SC and SG showed better mechanical properties: TS: 22.4 MPa TM: 3.65 GPa FS: 52.6 MPa FM: 4.51 GPa The addition of sisal fiber to pure carbon composite decreases mechanical properties	[226]
Polypropylene	Coir/Coconut shell	Twin screw extruder and injection moulding Fiber/filler ratio = 1:0, 3:1, 1:1, 1:3 and 0:1	Reinforcement loading: 20 wt % With a hybrid ration of 1:1, TS and TM increased 8% and 50% compared to the references, respectively	[227]
Epoxy	Kenaf/Kevlar	Hand lay-up Three types of kenaf fiber: woven, UD, mat	Woven kenaf hybrid composite showed better mechanical properties compared to UD ^6^ and mat TS: 145 MPa TM: 3.37 GPa FS: 100.3 MPa IS: 51.41 KJ/m^2^	[228]
Epoxy	Hemp/Sisal	Hot press Different layering sequence of fibers	The non-hybrid composites showed superior tensile and flexural properties than the hybrid composite due to the low compatibility of sisal/hemp fibers	[177]
Epoxy	Kenaf/Kevlar	Hand lay-up followed by compression Treated woven kenaf with NaOH Layering sequence: 4-layer and 3-layer with a different skin layer	Reinforcement loading: 30 wt % Mechanical properties of hybrid composite with 4-layer improved: TS: 64.7 MPa TM: 5.29 GPa FS: 51.28 MPa FM: 2.74 GPa IS: 50.1 KJ/m^2^ Kevlar as a skin layer improved tensile and flexural properties, but kenaf as a skin improved IS	[178]
Epoxy modified with LENR ^7^	Kenaf/Glass	Glass/kenaf ratio = 1:1 Treatment of kenaf with NaOH	Fiber treatment and adding of LENR to the matrix improved the mechanical properties: FS: 68.1 MPa IS: 13.1 KJ/m^2^	[229]
Polyester	Kenaf/Glass	Hand lay-up and hydraulic cold press Kenaf/glass ratio = 3:7 Sandwich configuration with glass shell and kenaf core Three types of kenaf: non-woven random mat, UD twisted yarn, plain-woven	Reinforcement loading: 35 wt % UD and woven fibers had higher tensile and flexural properties, respectively: TS: 194.6 MPa FM: 291.6 MPa	[230]
Epoxy	Jute/Glass	Epoxy/jute/glass weight ratio = 69/31/0, 68/25/7 and 64/18/19	The addition of glass and jute fibers with a ratio of 64/18/19 showed the highest mechanical properties: TS: 56.68MPa FS: 28.81 MPa FM: 1.83 GPa IS: 5.49 J	[231]
Polyethylene	Oil palm fiber (OPF) and clay particles	Extrusion and injection molding Alkali treatment of OPF	Reinforcement loading: 25 wt % The 12.5:12.5 hybrid composite showed 11% and 49% improvement of tensile strength and modulus, respectively	[232]
Epoxy	Sugar palm fiber (SPF)/Glass	Hand lay-up Benzoylation treatment on SPF Glass fiber ratio: 30%, 50%, and 70wt %	Glass fiber ration of 70 wt % exhibited the best tensile properties: 55.7% and 50.5% improvement of TS and TM, respectively Benzoylation treatment improved adhesion of fibers/matrix	[233]
Polypropylene	Sisal fiber (SiF)/Cellulose nanocrystals (CNC)	Melt-blending followed by injection molding SiF/CNC loading (29:1, 27:3, 25:5, and 23:7 wt %)	Enhancement of matrix with MAPP ^8^ compatibilizer Hybrid composite with SiF/CNC (27:3 wt %) showed highest TS (47.02 MPa), TM (2.82 GPa) and IS (38.62J/m) with 30.87% and 14.81% increment of FS and FM respectively	[234]
Epoxy	Hemp/polyethylene terephthalate (PET)	Vacuum-infusion	The TS and FS of interwoven hemp/PET hybrid composites were 4% and 22% greater than woven hemp composites	[235]
Epoxy	Flax/Glass	Compression-molding machine Sandwich structure: outer layers of glass/epoxy and the core from Flax/Epoxy	UD hybrid composite [0_G_/0_F_] has a higher TS (408.25 MPa), TM (31.97 GPa), FS (591.25 MPa), and FM (39.84 GPa) compared to angle ply [0_G_/ ± 45_F_] hybrid composite and also flax/epoxy composite	[180]
Epoxy	Arenga pinnata fiber (APF)/polyester yarn (PET)	Lay-up Mg(OH)_2_ as a flame retardant (5 wt %) APF:PET ratio = 0:5, 20:5, 35:5 and 50:5 wt %	Mg(OH)_2_ as a flame retardant Hybrid composite with 20 wt % and 35 wt % APF had the highest TM (165.2 MPa) and TS (9.69 N/mm^2^), respectively Increasing the fiber loading to the 50 wt % decreased the tensile properties	[236]
Ethylene propylene diene monomer (EPDM) rubber	Kevlar fiber (KF)/Nano-silica (NS)	Roll milling followed by compression molding	TS, elongation-at-break, and TM values of EPDM significantly increased by hybridization with KF and NS: TS: 4.94 MPa TM:51.09 MPa	[237]
PLA	Coir fiber (CF)/PALF With alkaline treatment	Internal mixer followed by compression molding CF:PALF ratios = 3:7, 1:1 and 7:3 Fibers loading: 30 wt %	Hybrid composite with higher PALF, C3P7 (CF:PALF = 3:7) exhibited the highest tensile properties: TS: 30.29 MPa TM: 5.16 GPa However, the C1P1 hybrid composite presented the highest IS	[238]

^1^ Tensile strength; ^2^ Flexural strength; ^3^ Impact strength; ^4^ Tensile modulus; ^5^ Flexural modulus; ^6^ Unidirectional; ^7^ Liquid epoxidized natural rubber; ^8^ Maleic anhydride grafted PP.

**Table 7:** Water absorption behavior of recently developed hybrid biocomposites.

Matrix	Fibers	Manufacturing Process	Treatment	Water Absorption	Ref.
PLA	Kenaf fiber and MMT clay	Extrude, roll mill compression molding	NaOH treatment	Adding one wt %, MMT decreased WA ^1^ due to the barrier effects	[261]
Green epoxy	Sisal/hemp	Hand lay-up and hot press	-	Higher WA of hybrid composite (12%) than the pure or non-hybrid composites (7%)	[177]
PLA	Aloevera fiber and MMT clay filler	Twin-screw extruder, two-roll mill, and compression molding method	NaOH treatment	Hybridization increased WA Increasing MMT content (3 wt %) maximized water-resistance of hybrid biocomposite	[262]
Epoxy	Luffa/coir/SiO_2_ nanospheres	Conventional molding Fiber loading: 40 vol %	NaOH treatment	Decrease of WA to 0.14% by adding 3 vol % of SiO_2_	[263]
polyester	Jute/glass Sisal/glass Sisal/jute/glass	Hand lay-up	Treatment with succinic anhydride and phthalic anhydride	Hybridization of JF and SF with glass fiber reduced the WA content significantly	[274]
Epoxy resin araldite	Sisal/coir (1:1)	Cold pressing	-	WA of hybrid composites increased with an increase of fiber wt % and soaking duration	[275]
Epoxy Resin	pineapple/coir (1:1)	Hand lay-up	-	Coir/pineapple/coir (CPC) pattern showed the highest water resistance to PCP and P/C patterns	[276]
Isothalic polyester	Jute/glass	Hand lay-up	UV radiation treatment	Improvement in water/moisture absorption resistance	[29]
Liquid diglycidyl ether of Bisphenol-A blended with neem oil	Kenaf fiber and sea-urchin spike filler	Casting	Amino silane surface treatment	Surface-modified kenaf fiber improved water resistance The addition of neem oil into epoxy did not change WA	[277]
Polypropylene	Sisal/glass	Injection molding	NaOH treatment	The addition of 10 and 20 wt % glass fibers showed improvement in water resistance	[278]
Epoxy resin	Hemp/jute Hemp/flax Hemp/jute /flax	Hand lay-up compression technique	-	Hemp/jute/epoxy, hemp/jute/flax/epoxy and hemp/flax/epoxy absorbed 4.5%, 3% and 2.8% water respectively	[240]
Low-density polyethylene	Jute/bamboo (1:1) cellulose Untreated jute/bamboo	Hot press	Dewaxing, alkaline treatment, and mercerization	Lower WA of treated cellulose hybrid composites (0.7%) with ten wt % fibers loading respect to untreated fiber	[279]
Starch-glycerol	Jute with and without epoxy resin coating (Araldite CY-230)	Wet hand lay-up and compression molding	Treatment by alkaline sodium hydroxide	A thin coating of epoxy reduced the WA significantly	[280]
Novolac type Phenolic formaldehyde	PALF/kenaf	Hot press	Triethoxy (ethyl) silane treatment	Treated hybrid composites revealed a reduction in WA 70P:30K ratio showed the lowest WA	[281]
Unsaturated polyester (UP) blended epoxy	E-glass fiber and iron (III) oxide particles	Hand lay-up	Amino-silane (APTMS) treatment	Low WA was observed for composites consist of 5 and 10 vol % of UP into epoxy	[282]
Epoxy	Coir fiber with Al_2_O_3_ filler	Hand-lay-up Fiber loading: 5, 10, 15, and 20 wt % Filler loading: 10 wt %	-	Amount of WA increased along with increasing fiber loading and decreasing epoxy loading	[283]
Thermoplastic SPS/agar (TPSA)	Sugar palm starch (SPS)	Hot press	-	Minimal improvement of water resistance properties	[219]
Epoxy LY 556	Date Palm Leaf (DPL)/glass	Hand lay-up	Alkaline treatment	The rate of WA of the composites increased by adding more DPL fiber Maximum water uptake in 30 wt % of DPL	[284]
Polypropylene	Banana/Coir	Twin-screw extruder and injection moulding Fiber loadings (CF/BF/PP): 15/5/80, 10/10/80, and 5/15/80 wt %	-	Increase of WA with an increase of soaking time and coil wt %	[222]
Epoxy	Sisal/Jute	Hand lay-up Jute/sisal ratio = 1:0, 1:3, 1:1 and 0:1	Alkaline treatment	Fiber loading: 30 wt % Lower WA of 1:1 hybrid composite due to the better interfacial bonding of matrix/fibers	[225]
Epoxy	Kenaf/Kevlar	Hand lay-up Three types of kenaf fiber: woven, UD, mat	-	Woven and UD kenaf absorbed less water	[228]
Epoxy	Jute/Glass	Epoxy/jute/glass weight ratio = 69/31/0, 68/25/7, and 64/18/19 wt %	-	THE lowest WA was for hybrid composite with a 64/18/19 ratio (11.7% after 1172 h immersion in water)	[231]
Epoxy	Hemp/polyethylene terephthalate (PET)	Vacuum-infusion	-	WA of the hemp/PET hybrid composite was half of the woven hemp composites	[235]
Epoxy	Flax/Glass	Compression-molding machine Sandwich structure: outer layers of glass/epoxy and the core from Flax/Epoxy	-	Hybrid composite of UD flax/glass/epoxy had a lower WA (4.6%) after 40 days to the carbon/epoxy and carbon/flax/epoxy composites	[180]
Vinyl ester	Flax/Basalt	Vacuum-infusion Fiber stacking sequence: BFFFFB	-	Hybrid composite exhibited lower WA compared to the flax/vinyl ester composite	[285]
Epoxy	Sugar palm fiber (SPF)/Glass	Hand lay-up Glass fiber ratio: 30%, 50%, and 70 wt %	Benzoylation treatment on SPF	Treated hybrid composite with 50wt % glass fiber exhibited min WA after 2h (0.16%)	[233]
PLA	Coir fiber (CF)/PALF	Internal mixer followed by compression molding CF:PALF ratios = 3:7, 1:1 and 7:3	Alkaline treatment	Fibers loading: 30 wt % C7P3 (CF:PALF = 7:3) showed the lowest WA (5.24%) after 7 days	[238]
PLA-g-GMA	Agave fibers/nanoclay particles	Extrusion	Compatibilizer: glycidyl methacrylate (GMA)	Compatibilizing compensated the hydrophilic character of agave fibers and decreased the WA	[206]
Unsaturated polyester	Sugar palm yarn/glass	Sheet molding process and hot press	-	Increasing the glass fiber content from 30% to 50 wt % improved WA properties	[213]

^1^ Water absorption.

**Table 8:** Flammability behavior of recently developed hybrid biocomposites.

Matrix	Fibers	Manufacturing Process	Flammability	Ref.
Novolac type Phenolic formaldehyde	PALF/kenaf Triethoxy (ethyl) silane treatment	Hot press	Phenolic resin formed a protective layer of char on the surface of composites. Combustion rates of the untreated hybrid composite were higher than the treated one.	[281]
Epoxy	Sisal/coir	Cold pressing	Increasing the fibers wt % increased flammability. This hybrid biocomposite was not suitable where the fire response is a serious consideration.	[275]
PLA	Banana fiber and nanoclay fillers (3 wt %)	Melt blending technique followed by injection molding Silane treatment	Improvement of thermal stability and fire retardancy by nanoclays that produced char as a thermal barrier to reduce combustion rate.	[311]
Cardanol	Kenaf fibers with recycled carbon filler Alkali treatment with 2% NaOH	Compression molding	Hybridization of kenaf fibers with recycled carbon filler improved the thermal stability and flammability property.	[312]
Epoxy	Banana short fiber and Al (OH)_3_ filler	Hand lay-up	Incorporation of Al (OH)_3_ particles reduced the rate of propagation of flame.	[313]
Polypropylene	Bamboo/glass	Compression molding	19% reduction of heat release rate and increase in the thermal stability.	[314]
Polypropylene	Biochar/wool fibers	Melt blending followed by injection molding	Improving fire resistance properties: 5 wt % wool fiber caused a delay in the onset of ignition and the time to reach peak heat release rate.	[287]
Polypropylene	Kenaf fibers with exfoliated graphite nanoplatelets	Melt extrusion	Graphene nanoplatelets improved the flame retardancy of composites: the fire performance index enhanced, the time to ignition prolonged, and the fire growth index reduced.	[315]
Epoxy Resin	pineapple/coir (1:1)	Hand lay-up	Layering pattern of coir/pineapple/coir (CPC) had higher resistance to burning.	[276]
High crystalline block copolymer polypropylene	Kenaf/wool with ammonium polyphosphate as a flame retardant and with ultraviolet ray stabilizer and colorant combination (UVC)	Thermal blending followed by injection molding	Reduction of sustained and forced combustion of the composite. Improvement of material response to the fire hazard.	[316]
Epoxy	Bamboo/kenaf/nanoclay Nanoclay types: halloysite nanotube (HNT), montmorillonite (MMT), and organically modified MMT (OMMT)	Hand lay-up	Flame retardancy improved with the loading of all types of nanoclay (OMMT was the best one). Improvement in flame properties in terms of peak heat release rate, total heat release, fire growth rate index, and the maximum average rate of heat emission and smoke growth rate.	[317]
Polyester	Banana peduncle fiber (BPF) with aluminum hydroxide (AH) particles Fiber treatment with vinyltriethoxysilane (VTS) and 3-aminoproply triethoxysilane (APTES) solutions	Hand lay-up	The addition of 10 wt % AH:10 wt % BPF to polyester composite retarded its burning. The ignition time and end of burning time delayed by 22.94% and 13.15%, respectively. The total heat release rate decreased by 29.68%.	[318]
Epoxy	Kenaf with nano oil palm empty fruit bunch (OPEFB) filler (3 wt %)	Hand lay-up	Hybrid nanocomposites presented better (and satisfactory) flame retardancy properties in comparison to kenaf/epoxy composites.	[319]
Epoxy	Arenga pinnata fiber (APF)/polyester yarn (PET)	Lay-up APF:PET ratio = 0:5, 20:5, 35:5 and 50:5 wt %	Mg(OH)_2_ as flame retardant. Hybrid composite of APF35/PET5/E55 with 5 wt % Mg(OH)_2_ exhibited a lower burning rate.	[236]
Ethylene propylene diene monomer (EPDM) rubber	Kevlar fiber (KF)/Nano-silica (NS)	Roll milling followed by compression molding	By increasing the KF loading, he flame retardant properties enhanced. Hybridization of KF and NS increased the TTI ^1^ noticeably.	[237]
PLA	Coir fiber (CF)/PALF Alkaline treatment of fibers	Internal mixer followed by compression molding CF:PALF ratios = 3:7, 1:1 and 7:3	Fibers loading: 30 wt %. C1P1 and C7P3 (CF:PALF = 7:3) exhibited higher thermal stability and char content.	[238]

^1^ Time to ignition.
